# Implementation of an Electrosurgical Checklist in a Podiatry Unit in Relation to a Case of Inadvertent Burns during Hallux Valgus Surgery

**DOI:** 10.3390/reports6030043

**Published:** 2023-09-14

**Authors:** Antonio Córdoba-Fernández, María Dolores Jiménez-Cristino, Francisco Javier Mármol-García, Victoria Eugenia Córdoba-Jiménez

**Affiliations:** 1 Faculty of Nursing, Physiotherapy and Podiatry, Universidad de Sevilla, Avicena St. s/n, 41009 Sevilla, Spain; mjimenez45@us.es; 2 Podiatric Clínical Area, Universidad de Sevilla, Avicena St. s/n, 41009 Sevilla, Spain; franciscojmarmol@hotmail.es; 3 Independent Researcher, Dr. Fleming St. 13, Bajo B, Castilleja de la Cuesta, 41950 Sevilla, Spain

**Keywords:** electrosurgical injury, monopolar diathermy, burns, electrosurgical safety

## Abstract

Iatrogenic burns are unpleasant and sometimes difficult to explain to patients. Podiatric surgeons routinely use electrosurgical devices to cut and coagulate tissue during surgical procedures. Although advances in technology have made electrosurgery increasingly safer for patients and personnel, its use is still poorly understood by the surgical community, and the hazards associated with its use still exist presently. Human error, direct or indirect transfer of electricity to a conductive device, or device malfunction can cause serious adverse events, including burns, electrical shocks, and or fires. Here, we report a rare case of a 43-year-old man who suffered severe burns during hallux valgus surgery. The surgeon and the nursing staff did not notice any injuries during the surgical intervention. This unusual clinical case serves to highlight the importance of implementing protocols to prevent injuries related to the use of electrosurgery. Based on this report, a specific checklist was implemented to prevent adverse events related to electrosurgery in our podiatric surgery unit to reduce the risk of electrosurgical complications. The implementation of the checklist can be useful to help health professionals improve patient safety during surgery and avoid potential medico–legal liability claims.

## 1. Introduction

Electrosurgery is the most used energy source in orthopaedic and podiatric surgery. Since its introduction into surgical practice, surgeons have increasingly used electrosurgery. Despite the technical improvement in terms of the safety of the equipment used, human error, direct or indirect transfer of electricity to conductive devices, or device malfunction can cause adverse events. The exact incidence of electrosurgical complications is not known exactly. Injuries related to diathermy have probably been under-reported, and most complications are treated without knowing the cause. It is estimated that approximately two-thirds of thermal injuries may not be detected during the procedure [1,2,3,4].

The physical principle of electrosurgery is based on generating high-frequency alternating energy from a low-frequency electrical current. This energy achieves the cutting and coagulation effects in the tissues in which it is applied, derived from the thermal energy that is generated. There are two commonly used electrosurgical modalities, depending on the number of electric poles at the site, monopolar and bipolar diathermy. Both electro–surgical devices require two poles to complete an electric current. The main difference between the two types of current is the distance between the poles, which determines the power used. In the unipolar mode, the tip of the device is the active pole, while the second pole is the return electrode or grounding plate. In the monopolar mode, a continuous current is necessary to produce vaporisation of cellular water around the electrode. Efficient cutting requires the electrode to move slowly but continuously through the tissue, while with bipolar current both poles are part of the tip of the instrument. Monopolar diathermy devices have a high-power output needed to overcome the long distance between the poles because the human body is a relatively poor conductor of electric current. Bipolar devices have a lower power, between one third and one tenth of that of unipolar systems, so the energy generated is insufficient to cut the tissue and can only desiccate it [5,6].

In orthopaedic surgery, the monopolar mode is the most widely used modality due to its more appropriate current for use in mixed mode, providing good dissection with varying degrees of coagulation. However, monopolar electrosurgery requires considerable knowledge, understanding, and vigilance of the surgeon to avoid the hazards of unintentional thermal injuries [5,6,7]. We report a rare case of iatrogenic partial-thickness electrosurgical burns on three toes during hallux valgus surgery. This unusual clinical case serves to highlight the importance of implementing protocols to prevent injuries related to the use of electrosurgery. On purpose of the herein reported case, a specific checklist was implemented to prevent adverse events related to monopolar diathermy in our podiatric surgery unit.

## 2. Detailed Case Description

A 43-year-old man presented at the surgical service of the Podiatric Clinic Area of the Universidad de Sevilla. He referred to having a painful matarsophalangeal joint (MPJ) with loss of flexor capacity in the hallux and slight hyperesthesia in the tips of the hallux and in the second and third toes of the left foot. The patient did not mention significant medical–surgical history or systemic diseases, and the vascular examination showed the presence of distal pulses with normal capillary refill without signs of hypoperfusion in both feet. Doppler examination showed the presence of arterial flow of the tibialis and dorsalis pedis with normal ankle–brachial index test.

**Figure 1:**
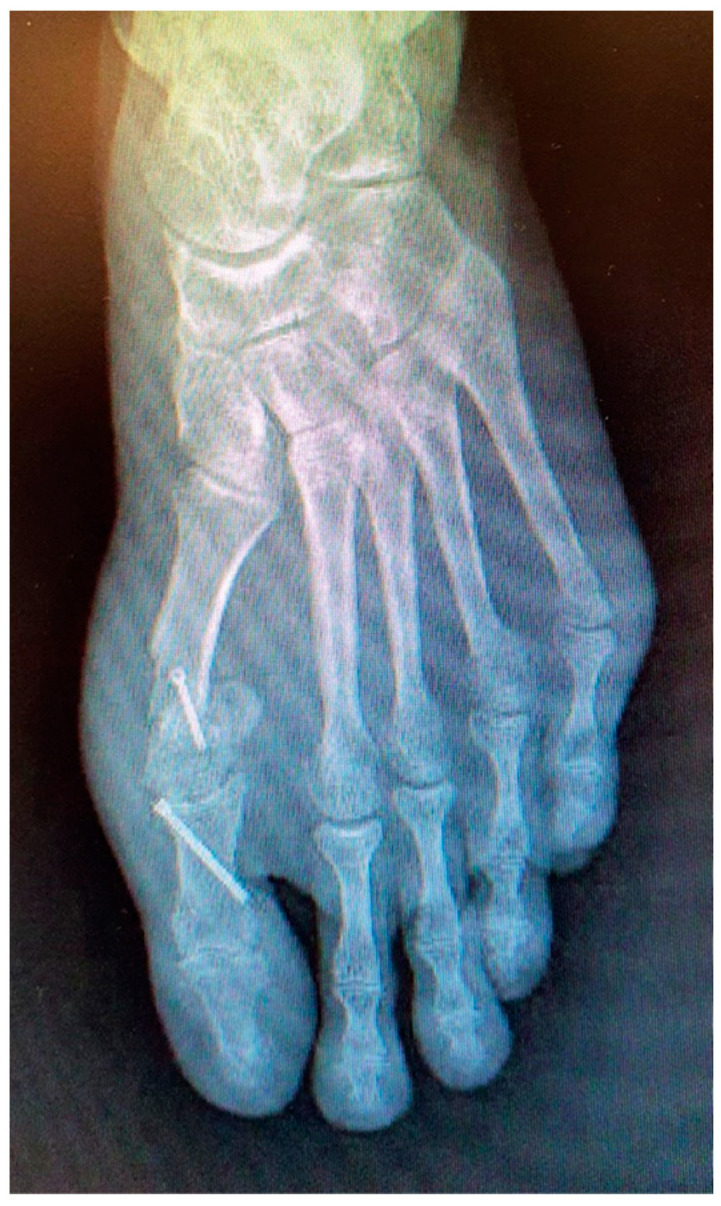
Postoperative radiological aspect of foot after the first procedure.

**Figure 2:**
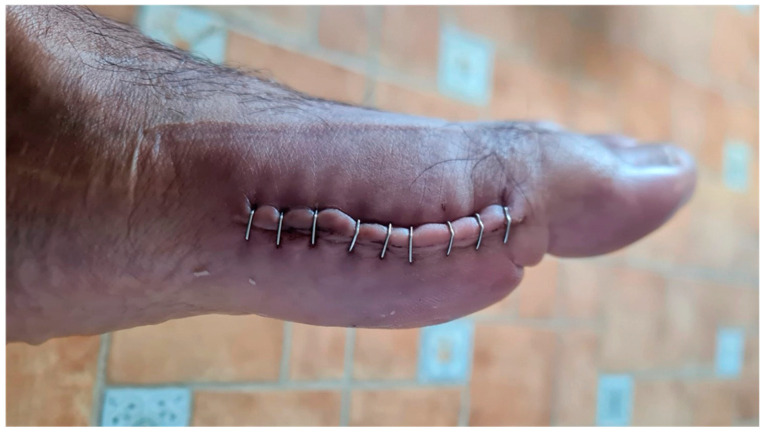
Postoperative appearance of the medial incision prior to staple removal.

In May 2021, he underwent an operation to correct the hallux valgus deformity using chevron capital osteotomy and a proximal Akin procedure (Figure 1). The patient reported that a year later he had to be operated on again due to pain at the level of the first MPJ to remove the osteosynthesis material. The second surgery was performed outpatiently with epidural anaesthesia through a medial incision at the MPJ level (Figure 2).

**Figure 3:**
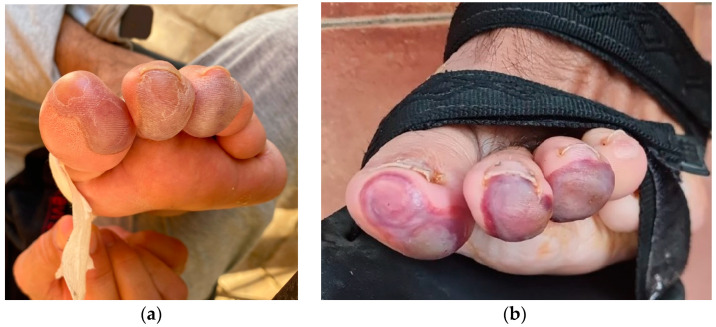
Appearance of the foot at seven days (**a**), and two weeks postoperatively (**b**).

**Figure 4:**
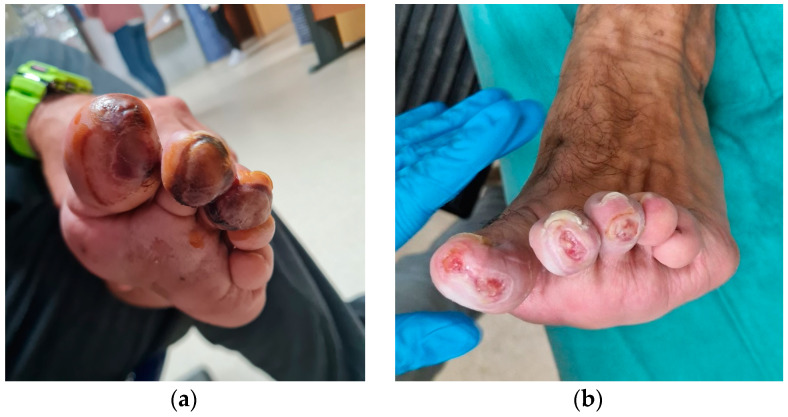
Rupture and desiccation of the blisters with areas of epidermal necrosis can be observed after three weeks (**a**). Aspect after surgical excision of epidermal necrosis areas (**b**).

The patient was reviewed to perform the first dressing change and surgical control a week after surgery, and the responsible nurse noticed blisters fade under pressure with pain in the ball of the first, second, and third toes compatible with partial-thickness burns (Figure 3a). The patient reported having suffered severe pain at the level of the toes in the operated foot and difficulty walking during the first three days after surgery. He reported that the pain did not subside with the prescribed medication (acetaminophen 650 mg every 8 h per os). After consulting with the surgeon, he was unaware of the circumstances that caused the injuries, and the nursing staff was urged to take care of the wounds for both the surgical incision and the burns. The injuries were initially treated by the patient at home with applications of povidone iodine antiseptic solution. After two weeks, the skin staples were removed from the medial incision, and adequate closure of the surgical incision could be verified. On the injured toes, the redness of the blisters was observed to fade under pressure (Figure 3b). At three weeks, rupture and desiccation of the blisters with epidermal necrosis areas were observed in the burns and surgical excision was performed. The local application of silver sulfadiazine was carried out every 48 hours until complete healing that was achieved at 7 weeks (Figure 4a,b).

**Figure 5:**
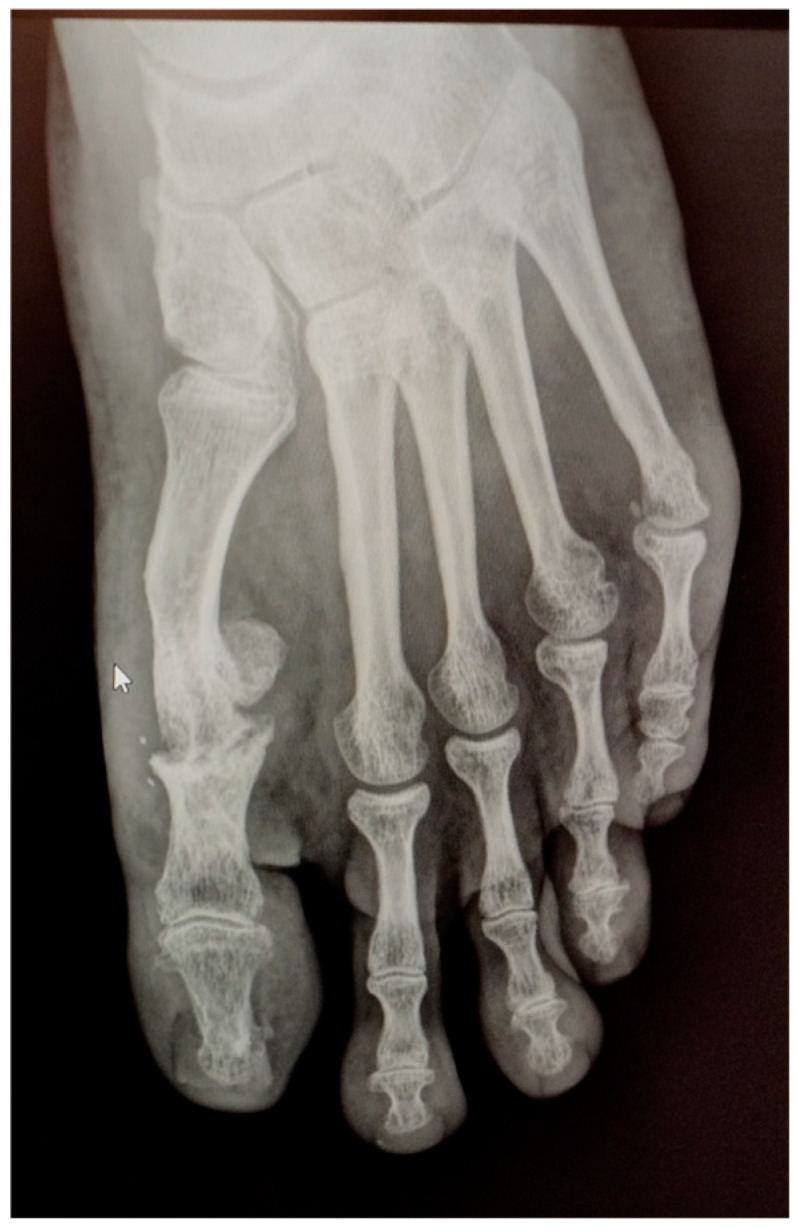
Postoperative radiography one year after the second procedure. Signs of severe joint destruction compatible with resection arthroplasty can be observed.

Radiographic examination after one year following the second surgery showed signs of severe joint destruction compatible with MPJ resection arthroplasty with significant shortening of the first toe (Figure 5). The patient was treated conservatively with orthopaedic insoles.

## 3. Discussion

Most adverse events related to electrosurgery are related to thermal energy and have been commonly reported as burns. Thermal injuries are those that are caused by inadvertent use of an active electrode in any part of the body apart from the intended organ or tissue. Indirect injuries are those that occur because of contact of the active electrode with any other metal instrument, which, in turn, is in contact with the tissue, or those injuries that occur outside the operating field due to the spread of current from the shaft of the active electrode to nearby tissue.

In orthopaedic surgery, the monopolar electrosurgery mode is the most widely used modality due to its most appropriate current for use in the mixed mode, providing good dissection with varying degrees of coagulation [5,6]. There are several drawbacks of monopolar electrosurgery; it requires considerable knowledge, understanding, and vigilance of the operator to avoid the hazards of unintentional thermal injuries. Accidental burns usually occur due to inadvertent contact with active or heated electrodes, direct or capacitive coupling, insulation defects in instruments or connections, and improper placement of the return electrode [5,6,7].

An American College of Surgeons survey showed that 18% of laparoscopic surgeons experienced a complication attributed to electrosurgery, and 70% of burns originating during laparoscopic surgery are estimated to be unidentified [3]. In a review (January 1994 to December 2013) conducted using the FDA database on Surgical Energy-Based Device Injuries, 178 deaths and 3553 injuries were reported. Most injuries caused by electrosurgery were commonly referred to as burns by direct application (32%), dispersive electrode burns due to ground failure (29%), and burns due to insulation failure (14%). Almost half of the reported complications (45%) occurred with monopolar electrosurgical devices [1].Inadvertent burns may occur in several ways during the use of electrosurgery. The reported burn injuries were most often caused by an improper application of a neutral electrode or by involuntary contact of the active electrode with tissue [8,9]. Thermal injuries due to device insulation failure of the device, direct coupling, or capacitive coupling are rare [10,11].

Capacitive coupling is a condition that occurs when electrical current is transferred from a conductor through intact insulation to adjacent conductive materials. Direct coupling occurs when another metal object such as a probe or retractor is touched by the active electrode. When the active electrode encounters another metal instrument, energy can be transferred from the active electrode to the instrument. In monopolar electrosurgery, direct coupling is often intentionally utilised to coagulate small bleedings using haemostatic forceps, which are held in contact with the active electrode. However, when the active electrode comes into unintended contact with another electrode or non-insulated conductive instrument, current from the active electrode flows through the adjacent instrument through the pathway of least resistance, and potentially damages adjacent structures or tissues not within the visual field that are in direct contact with the secondary instrument.

Adverse events caused by direct coupling have been widely described in the literature as a complication associated with laparoscopic surgery [4,7]. In orthopaedic surgery, most of the reported cases occurred during arthroscopic surgery procedures. Reported cases in open surgery are rarer [12,13,14]. In the present case, it is likely that the injuries were caused by direct coupling when the active electrode inadvertently encountered a non-insulated metallic instrument such as an orthopaedic retractor. The current could have been carried through the surgical instrument, causing burns to the tissue which was in contact with the retractor. Injuries could also have occurred if the metal retractor encountered a haemostat that was energised to coagulate a bleeding vessel.

The literature review shows that in 82% of the cases injuries from energy-based surgical devices were identified intraoperatively, in 9% postoperatively in inpatients, and in 9% after discharge [1]. In laparoscopic surgery, approximately two-thirds of the injuries may not be detected during the procedure [3,4]. As in the present case, the injuries were undetected and not recognised until the following five days when partial-thickness burns were observed on the skin between one or three inches away from the medial incision (MPJ), appearing far away from the region where the electrosurgery had beenperformed. These burn sites could be an area where the retractor rested on the skin.

To avoid accidents due to direct coupling, as reported in this case, the exact location of the active electrode must always be controlled when it is being energised. Special care should be taken whenever the active electrode is energised near another metal object, especially retractors. Given that the surgical approach was made through a medial incision, it is likely that the plantar location retractor was the one that repeatedly encountered the active electrode, causing burns in the plantar area of the toes.

Because metal is a conductor far superior to tissue, it is possible that current density can increase around the metal implant if it is located between the active and dispersive electrodes. To date, no case reports have been reported on alternate site burns related to orthopaedic implants. The most commonly used metal implants in orthopaedic surgery are made of titanium. The electrical conductivity of titanium is very low because the availability of two free electrons in a 3D orbital makes titanium conduct electricity, but it is always at a low intensity. Due to this circumstance, it is unlikely that in the present case the removal of the osteosynthesis material (screws) could be related to the origin of the burns. However, remote thermal injury caused by aberrant intraoperative current grounding through titanium plating implants has been reported [14]. When metallic implants cannot be removed prior to electrosurgery use, the best way to avoid this risk is to reduce the distance between the electrodes or, if necessary, use a bipolar device.

The available evidence shows limited training in diathermy in trainees with a lack of awareness among surgeons which results in a failure to adhere to what is considered best practice [15,16,17]. The role of all the personnel involved in electrosurgical practice is critical in the implementation of precautions to prevent electrosurgical injuries. Staff training, along with regular safety inspections, and the implementation of a standardised process are key to minimizing such risks and injuries associated with the use of electrosurgical devices.

**Figure 6:**
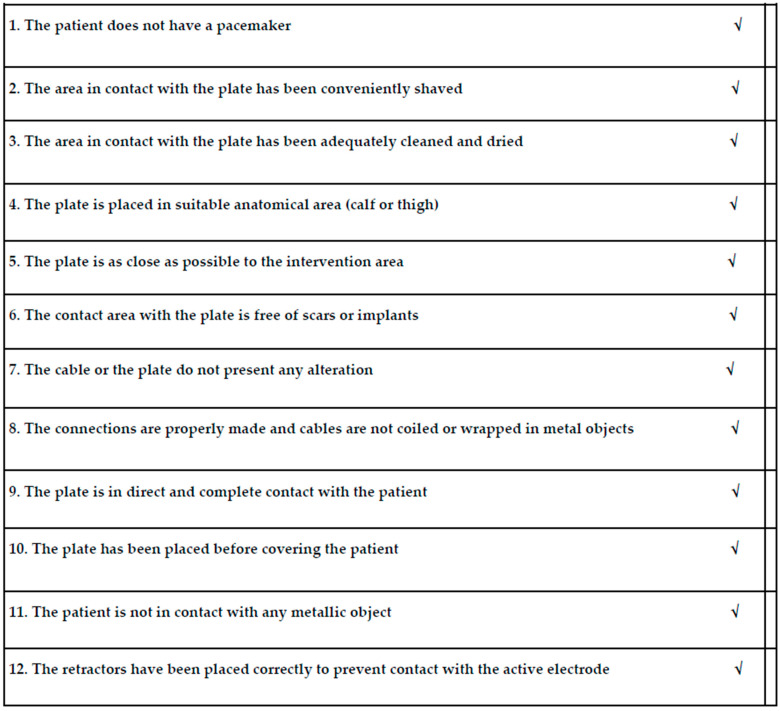
Electrosurgical safety checklist model. Items to evaluate before the use of monopolar diathermy.

The incidence of error in surgery such as those reported here should be reduced, and surgeons and perioperative nurses should standardise processes and preoperatively assess risks, including electrosurgical injuries. The Surgical Safety Checklist is a useful tool developed by the World Health Alliance for Patient Safety; to help health professionals improve patient safety during surgery [18]. Numerous specialities have incorporated checklists in their strategies for the safety of surgical interventions [19]. However, nowadays it is necessary to continue to reduce the incidence of errors in surgery. The Guideline for Electrosurgical Safety provides guidance to perioperative personnel on the safe use of electrosurgical units [20]. Regarding the case presented, and to adapt and implement a tool based on clinical practice guidelines related to electrosurgical safety, we have developed a specific checklist model to improve safety related to possible adverse effects associated with the use of monopolar diathermy in our podiatry unit (Figure 6).

## 4. Conclusions

In conclusion, iatrogenic burns are unpleasant and sometimes difficult to explain to the patient, as in the case presented here. We consider that the risk of thermal injuries with monopolar electrosurgery has been neglected by the surgical community for decades. The implementation of the checklist can be certainly useful to help health professionals improve patient safety during surgery and avoid potential medico–legal liability claims.

## Data Availability

All data generated during this study are included in this article. Further enquiries can be directed to the corresponding author.
